# Clonal structure, resistome and virulence gene profiles of methicillin-resistant *Staphylococcus aureus* from retail chicken meat in the United Arab Emirates

**DOI:** 10.3389/fmicb.2026.1817264

**Published:** 2026-04-13

**Authors:** Ihab Habib, Syrine Boucherabine, Glindya Bhagya Lakshmi, Stefan Monecke, Ralf Ehricht, Richard Goering, Fatima Al Dhaheri, Mushtaq Khan, Abiola Senok

**Affiliations:** 1 Veterinary Public Health Research Laboratory, College of Agriculture and Veterinary Medicine, United Arab Emirates University, Al Ain, United Arab Emirates; 2 ASPIRE Research Institute for Food Security in the Drylands (ARIFSID), United Arab Emirates University, Al Ain, United Arab Emirates; 3 College of Medicine, Mohammed Bin Rashid University of Medicine and Health Sciences, Dubai, United Arab Emirates; 4 Department of Biological Sciences, College of Medicine and Health Sciences, Khalifa University, Abu Dhabi, United Arab Emirates; 5 Leibniz Institute of Photonic Technology (IPHT), Leibniz Center for Photonics in Infection Research (LPI), Jena, Germany; 6 InfectoGnostics Research Campus, Centre for Applied Research, Jena, Germany; 7 Institute of Physical Chemistry, Friedrich Schiller University, Jena, Germany; 8 Center for Translational Medicine (CETRAMED), Jena University Hospital, Friedrich Schiller University Jena, Jena, Germany; 9 Department of Medical Microbiology and Immunology, Creighton University School of Medicine, Omaha, NE, United States; 10 Department of Pediatrics, College of Medicine and Health Sciences, United Arab Emirates University, Al Ain, United Arab Emirates; 11 Zayed Bin Sultan Centre for Health Sciences, United Arab Emirates University, Al Ain, United Arab Emirates; 12 Department of Medical Microbiology and Immunology, College of Medicine and Health Sciences, United Arab Emirates University, Al Ain, United Arab Emirates; 13 School of Dentistry, Cardiff University, Cardiff, United Kingdom

**Keywords:** antimicrobial resistance, DNA microarray, methicillin-resistant *Staphylococcus aureus*, one health, retail chicken meat

## Abstract

Methicillin-resistant *Staphylococcus aureus* (MRSA) is an important public health hazard at the human–food–environment interface. Poultry meat has been increasingly implicated as a potential source of MRSA exposure, yet molecular data from the Middle East remain scarce. This study aimed to characterize MRSA isolated from retail chicken meat in the United Arab Emirates (UAE) using a DNA microarray platform and to compare these profiles with recent UAE clinical MRSA data. A total of 34 non-duplicate MRSA isolates recovered from retail chicken meat were analyzed using a high-resolution DNA microarray targeting clonal complexes, SCC*mec* elements, antimicrobial resistance genes and virulence determinants. Isolates were assigned to six clonal complexes, with CC5 (18/34, 52.9%) and CC6 (12/34, 35.3%) predominating, while CC1, CC22, CC88 and CC97 each occurred at low frequency. Most isolates carried community-associated SCC*mec* types IV or V/VT. All (100%) harbored the *mecA* gene, whereas resistance genes associated with fusidic acid (*fusC*), macrolide–lincosamide resistance (*ermC*), tetracycline resistance (*tetM* and/or *tetK*), phenicol resistance (*fexA*) and fosfomycin resistance (*fosB*) were detected in 14/34 (41.2%), 15/34 (44.1%), 20/34 (58.8%), 18/34 (52.9%) and 30/34 (88.2%) isolates, respectively, while no *vanA* or *vanB* genes were identified. Virulence profiling revealed widespread carriage of immune evasion cluster genes, including *sak* (32/34, 94.1%) and *scn* (30/34, 88.2%), together with universal detection of major adhesion and biofilm-associated genes. Panton–Valentine leukocidin genes (*lukF-PV/lukS-PV*) were present in 2/34 (5.9%) isolates and the toxic shock syndrome toxin gene (*tst1*) in 1/34 (2.9%). A diverse repertoire of staphylococcal enterotoxin genes was observed, with enterotoxin gene cluster components (e.g., *seg*, *selm*, *selu*) detected in up to 19/34 (55.9%) isolates and the classical enterotoxin gene *sea* in 12/34 (35.3%) isolates. Overall, these findings indicate that MRSA contaminating retail chicken meat in the UAE is dominated by human-associated, community-type lineages with extensive antimicrobial resistance and virulence gene content, paralleling recent clinical MRSA clonal structures in the country. This study provides the first microarray-based molecular characterization of MRSA from retail chicken meat in the UAE and supports integration of food-chain surveillance into national One Health MRSA monitoring efforts.

## 1 Introduction

Methicillin-resistant *Staphylococcus aureus* (MRSA) remains a major contributor to the global antimicrobial resistance (AMR) burden, causing healthcare-associated and community-acquired infections and associated with increased morbidity, mortality and healthcare costs (Algammal et al., 2020; Pal et al., 2024). Resistance to β-lactam agents is mediated primarily by acquisition of *mecA* (or *mecC*) located on staphylococcal cassette chromosome *mec* (SCC*mec*), enabling MRSA to withstand most penicillins and cephalosporins and complicating the treatment of serious infections (Benkerroum, 2018; Nunes Nascimento et al., 2026). From a One Health perspective, MRSA exemplifies the interconnectedness of human, animal and environmental health. *S. aureus* colonizes the skin, nasopharynx and gastrointestinal tract of humans and multiple animal species, including poultry, permitting circulation of strains and mobile genetic elements across compartments (Abdullahi et al., 2021; Thacharodi et al., 2026).

Meat and meat products are recognized as potential vehicles for MRSA exposure, especially when handling raw meat or consuming undercooked products (Hanson et al., 2011; Wendlandt et al., 2013). A recent systematic review and meta-analysis estimated a pooled MRSA prevalence of 9.13% in meat and meat products in the Eastern Mediterranean region, underscoring the relevance of foodborne MRSA for this part of the world (Xing et al., 2025). Poultry and poultry meat have increasingly been implicated in the epidemiology of MRSA (Ribeiro et al., 2018; Xing et al., 2025). Both human-associated lineages (e.g., CC5, CC8, and CC22) and livestock-associated MRSA (LA-MRSA) lineages such as CC398 and CC9 have been described in poultry production and along the farm-to-fork pathway (Ribeiro et al., 2018; Fetsch et al., 2021; Silva et al., 2021). In the Middle East and North Africa (MENA) region, MRSA has been reported in retail poultry meat from Egypt, Saudi Arabia, Iran and Algeria, often with substantial carriage of antimicrobial resistance (AMR) determinants and staphylococcal enterotoxins (Sallam et al., 2015; Raji et al., 2016; Rahimi and Shafiei, 2019; Achek et al., 2021; Abolghait et al., 2020).

In the United Arab Emirates (UAE), national AMR surveillance data show that MRSA accounts for roughly one-quarter of all *S. aureus* isolates associated with human clinical infections, with the proportion of MRSA increasing over time (Thomsen et al., 2023; Senghore et al., 2026). Data on MRSA in the animal and food sectors in the UAE remain limited. A recent retail food survey in the UAE showed that MRSA was detected in 75% of chicken meat samples, compared with lower frequencies in beef, lamb and camel meat, and also identified MRSA in fresh-cut fruit and vegetables (Habib et al., 2025a). Despite the high MRSA prevalence in chicken meat, no in-depth characterization has been done to study clonal, resistome and virulence profile for poultry-associated MRSA isolates in the UAE. DNA microarray genotyping has proven to be a useful tool for MRSA surveillance across clinical, animal and environmental settings, enabling simultaneous assignment of CC/strain type and detection of a wide panel of resistance and virulence determinants (Senok et al., 2020; Boucherabine et al., 2025; Achek et al., 2021). In the UAE, microarray-based typing has yielded lineage and gene profiles that closely mirror WGS results, while being quicker and more operationally feasible for routine surveillance (Senok et al., 2020; Boucherabine et al., 2025).

Given that chicken meat showed the highest MRSA prevalence among tested food categories and represents a major component of the Emirati diet, detailed molecular characterization of chicken-derived MRSA is a priority for food safety and One Health AMR surveillance (Habib et al., 2025a). This study aimed to provide the first DNA microarray-based molecular characterization of MRSA isolated from retail chicken meat in the UAE, and to determine whether these isolates predominantly belonged to human-associated clonal lineages mirroring those found in recent UAE clinical collections. We analyzed clonal complexes, SCC*mec* types, antimicrobial resistance genes and virulence determinants and compared these molecular profiles with recent UAE clinical MRSA microarray data. By integrating food-chain and clinical datasets, this work generates novel baseline evidence to distinguish human-associated from livestock-associated MRSA signals in poultry meat and thereby strengthens the One Health understanding of MRSA at the human–food interface in the UAE.

## 2 Materials and methods

### 2.1 Sampling context and bacterial isolates

The MRSA isolates characterized in this study originated from a previously conducted baseline survey investigating MRSA occurrence in retail food products in Dubai, UAE (Habib et al., 2025a). In that survey, 260 food samples representing multiple categories (red meat, poultry, camel meat, and fresh-cut fruit and vegetables) were collected from retail outlets across Dubai using a cross-sectional design. Detailed sampling procedures, laboratory methods and antimicrobial susceptibility results have been reported elsewhere (Habib et al., 2025a); from which the present study draws a subset of MRSA isolates for additional molecular characterization. Of the 45 MRSA isolates recovered in the original survey (Habib et al., 2025a), 34 were selected for microarray analysis as an epidemiologically independent subset, excluding repeated isolates from the same batch and retaining representation of distinct samples and sources. This selection strategy was intended to reduce redundancy rather than to enrich for specific lineages, thereby supporting a more reliable interpretation of clonal distribution.

Briefly, retail chicken meat samples (*n* = 60) were obtained from supermarkets and hypermarkets distributed across different areas of Dubai. Samples were transported to the laboratory under chilled conditions and processed within 24 h of purchase. Detection and isolation of presumptive MRSA were performed using selective chromogenic media, followed by incubation at 35–37 °C, as described by Habib et al. (2025a). Colonies consistent with *S. aureus* morphology were subcultured and subjected to species-level identification using matrix-assisted laser desorption/ionization time-of-flight mass spectrometry (MALDI-TOF MS) (Habib et al., 2025a). Molecular confirmation of *S. aureus* and methicillin resistance was undertaken using multiplex PCR assays targeting *nuc* and *mecA*, respectively, following the methods of Perez-Roth et al. (2001).

Antimicrobial susceptibility testing for a panel of clinically relevant agents was performed using the VITEK^®^ 2 automated system (bioMérieux, Marcy-l’Étoile, France) with the AST-P592 card, and resistance patterns were interpreted according to contemporary clinical breakpoints; full phenotypic resistance data are presented in Habib et al. (2025a). Out of the 60 chicken meat samples, MRSA was recovered from 45 samples (75.0%) (Habib et al., 2025a). For the purposes of the present microarray-based characterization, a subset of 34 non-duplicate MRSA isolates was selected to ensure that each isolate originated from a different retail chicken meat batch. These 34 isolates were stored at −80 °C in glycerol stocks and later revived for DNA microarray genotyping.

### 2.2 DNA microarray-based genotyping

**Figure 1:**
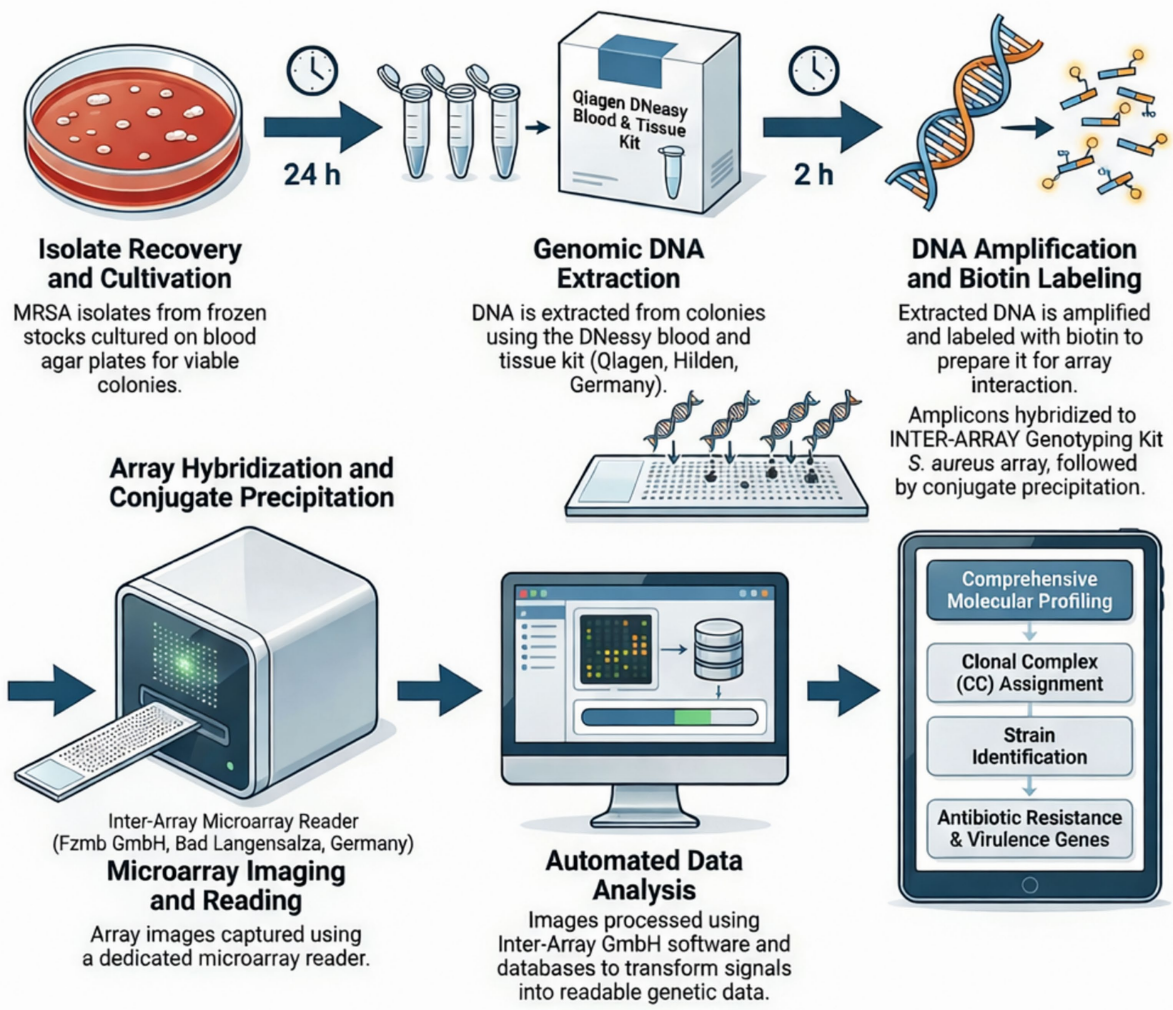
Illustration of the systematic laboratory workflow of the DNA microarray assay designed to identify and analyze methicillin-resistant *Staphylococcus aureus* from retail chicken meat in the United Arab Emirates.

Figure 1 depicts the systematic methodology workflow utilized in this study. Molecular characterization of MRSA isolates was performed using the INTER-ARRAY *S. aureus* Genotyping Kit (Inter-Array GmbH, Bad Langensalza, Germany), following manufacturer instructions and procedures described in previous microarray-based MRSA studies from the UAE (Senok et al., 2020; Boucherabine et al., 2025). Isolates were retrieved from frozen stocks and streaked onto blood agar plates, then incubated at 37 °C for 24 h to obtain fresh colonies.

Genomic DNA was extracted from overnight cultures using the DNeasy Blood and Tissue Kit (Qiagen, Hilden, Germany) according to the manufacturer’s protocol. Purified DNA was subjected to amplification and biotin labelling using the INTER-ARRAY kit reagents (Inter-Array GmbH, Germany). The labelled amplicons were hybridized to the *S. aureus* microarray, which comprises probes for species markers, SCC*mec* elements, clonal complex markers and a broad panel of antimicrobial resistance and virulence genes (Monecke et al., 2018; Senok et al., 2020; Boucherabine et al., 2025).

Following hybridization and washing, the microarrays were scanned using the Inter-Array microarray reader (Inter-Array GmbH, Germany). Signal patterns were interpreted using dedicated Inter-Array software and reference databases, which facilitate automated assignment of isolates to clonal complexes and strain types, as well as qualitative detection of target resistance and virulence genes (Monecke et al., 2018; Achek et al., 2021; Boucherabine et al., 2025).

For each isolate, the following data were recorded: (i) clonal complex (CC) and strain assignment; (ii) SCC*mec* type; and (iii) presence or absence of *mecA*, *blaZ*, *blaI*, *blaR*. Additional antimicrobial resistance determinants were captured, including *erm* genes (*ermA*, *ermC*), *linA*, *msrA*, aminoglycoside resistance genes (*aacA*, *aadD*, *aphA3*, *sat*), tetracycline resistance genes (*tetM*, *tetK*), phenicol resistance determinants (*fexA*, *cat*), the fosfomycin resistance gene *fosB* (and *fosBfusC*, *far1*), vancomycin resistance genes (*vanA*, *vanB*) and other resistance markers included in the array. Virulence (*lukF*, *lukS*, *tst1*), immune evasion cluster genes (*sak*, *chp*, *scn*), adhesion and biofilm (*sasG*, *clfA*, *clfB*, *fnbA*, *fnbB*, *icaA*, *icaC*, *icaD*, *bap*, *cna*), exfoliative toxin genes (*etA*, *etB*, *etD*), ACME, and a panel of staphylococcal enterotoxin and enterotoxin were also captured.

### 2.3 Quality control and validation of microarray analysis

Quality control of the INTER-ARRAY *S. aureus* Genotyping Kit (Inter-Array GmbH, Bad Langensalza, Germany) comprised both batch-level validation and run-level assessment. At the batch level, array performance was verified by confirming correct probe deposition and positioning and by ensuring that blank positions did not generate false-positive hybridization signals. External controls, including non-template controls and non-*S. aureus* DNA controls, were used to monitor background signals and detect non-specific reactivity or systematic technical issues. In addition, a panel of fully sequenced reference strains with known genotypes and expected hybridization patterns, including strains such as MW2 and N315, was used to assess probe behavior and concordance between observed and expected results (as per manufacturer protocol; Inter-Array GmbH, Bad Langensalza, Germany).

At the run level, each individual experiment was evaluated for validity based on the detection of species-specific markers included in the assay. Experiments with absent or weak species-marker signals were considered invalid. The complete hybridization profile was further assessed using the accompanying analysis software for plausibility against established reference patterns and previous validated experiments, allowing recognition of failed runs, atypical profiles, mixed cultures, or other technical artefacts. Only experiments fulfilling these internal validity criteria were accepted for interpretation.

To reduce the likelihood of false-negative calls for individual targets, probes were designed against conserved regions whenever possible, each probe was spotted in duplicate at different positions on the array surface, and, for selected resistance and virulence determinants or gene clusters, multiple probes targeting distinct regions were included (Inter-Array GmbH, Bad Langensalza, Germany). These combined measures were used to support the reliability of both positive and negative microarray results.

### 2.4 Data handling and analysis

Microarray outputs were exported to spreadsheet format for descriptive analysis. Clonal complex distribution and strain assignments were summarized as counts and percentages. Resistance and virulence gene frequencies were expressed as the number and percentage of isolates carrying each gene. Data were tabulated to enable cross-comparison of CC/strain background with SCC*mec* types, resistance determinants and virulence profiles. No inferential statistics were applied, as the study objective was to provide a descriptive baseline molecular characterization of MRSA from retail chicken meat.

## 3 Results

### 3.1 Clonal complexes, strain assignments, and SCC*mec* types

All 34 MRSA isolates recovered from retail chicken meat were successfully characterized using DNA microarray-based analysis. The isolates were assigned to six distinct clonal complexes (CCs). CC5 was the most prevalent CC, comprising 18/34 isolates (52.9%), followed by CC6 with 12/34 isolates (35.3%). The remaining CCs—CC1, CC22, CC88 and CC97—were each represented by a single isolate, together accounting for 4/34 isolates (11.8%) (Table 1).

**Table 1:** Distribution of clonal complexes and corresponding strain classifications among MRSA isolated from retail chicken meat in the United Arab Emirates.

Clonal complex	Strain assignment	Number of strains
CC1 (*n* = 1)	CC1-MRSA-[V/VT + *fusC+ccrAB1*]	1
CC5 (*n* = 18)	CC5-MRSA-V/VT (*sed*/j/r+), WA MRSA-11/34/35/87/90/108	1
	CC5-MRSA-[VI + *fusC*]	8
	CC5-MRSA-[V/VT + *fusC*] (*sed*/j/r+),	5
	CC5-MRSA-V/VT (*sed/j/r*-), WA MRSA-81/85/86/123	4
CC6 (*n* = 12)	CC6-MRSA-IV, WA MRSA-51	12
CC22 (*n* = 1)	CC22-MRSA-IV (PVL+/*tst*+)	1
CC88 (*n* = 1)	CC88-MRSA-V/VT (PVL+), WA MRSA-117	1
CC97 (*n* = 1)	CC97-MRSA-IV, WA MRSA-54/63	1

Within CC5, the microarray identified several strain variants that differed in SCC*mec* type and accessory gene composition. Most CC5 isolates carried SCC*mec* type V or VT, while a smaller subset carried SCC*mec* type IV. Of the CC5 isolates, 12/18 carried the fusidic acid resistance gene *fusC* and components of enterotoxin gene clusters, while 6/18 lacked these elements (Table 1). In contrast, all CC6 isolates were assigned to a single strain designation, CC6-MRSA-IV (WA MRSA-51), showing a high degree of genetic homogeneity within this clonal complex.

Isolates belonging to CC1, CC22, CC88 and CC97 each exhibited unique strain assignments. The CC1 isolate carried PVL genes (*lukF-PV/lukS-PV*), while the CC88 isolate harboured *tst1* (Table 1). Overall, SCC*mec* types IV and V/VT predominated across the collection. No SCC*mec* II or III elements were detected (Table 1). Overall, SCC*mec* types IV and V/VT predominated across the collection, consistent with community-associated MRSA backgrounds. No SCC*mec* II or III elements were detected (Table 1).

### 3.2 Antimicrobial resistance gene profiles

The distribution of antimicrobial resistance genes among the 34 MRSA isolates is summarized in Table 2. All isolates (34/34, 100%) harboured *mecA*, confirming methicillin resistance at the genetic level. Genes associated with β*blaZ*, *blaI* and *blaR* were detected in 27/34 isolates (79.4%) (Table 2).

**Table 2:** Detection of antibiotic resistance genes among MRSA isolated from retail chicken meat in the United Arab Emirates.

Antibiotic resistance genes		No. positive	% positive
*mecA*	Alternate penicillin binding protein 2a	34	100
*merA; merB*	Mercury resistance operon	0	0
*blaZ;blaI;blaR*	Beta-lactamase operon	27	79
*ermA*	R-RNA adenine n-6-methyl-transferase, Erythromycin/clindamycin resistance	0	0
*ermB*	Erythromycin/clindamycin resistance	1	3
*ermC*	Erythromycin/clindamycin resistance	15	44
*linA*	Lincosamide nucleotidyltransferase	0	0
*msrA*	energy-dependent efflux of erythromycin	1	3
*vatB*	Acetyl-transferase inactivating streptogramin A	0	0
*vgaA*	ATP binding protein, streptogramin A resistance	0	0
*aacA-aphD*	Bifunctional enzyme gentamicin resistance	4	12
*aadD*	Aminoglycoside adenyl-transferase, tobramycin resistance	4	12
*aphA3*	3′5′-aminoglycoside phosphotransferase, neo−/kanamycin resistance	1	3
*sat*	Streptothricin acetyltransferase	1	3
*dfrA*	Dihydrofolate reductase type 1	6	18
*far1*	Fusidic acid resistance (plasmid-borne)	0	0
*Q6GD50 (fusC)*	SCC-associated fusidic acid resistance	14	41
*mupA*	Mupirocin resistance protein	0	0
*tetK*	Tetracycline resistance	2	6
*tetM*	Tetracycline resistance	18	53
*cat*	Chloramphenicol acetyltransferase	0	0
*fexA*	Chloramphenicol/florfenicol exporter	18	53
*fosB*	Metallothiol transferase	30	88
*fosB-plasmid*	Metallothiol transferase	0	0
*qacA; qacC*	Quaternary ammonium compound resistance protein A/B	2	6
*tetEfflux*	Transport−/efflux protein	31	91
*vanA; vanB*	Vancomycin resistance genes	0	0

Macrolide–lincosamide resistance determinants were variably present. The *ermC* gene, encoding a ribosomal RNA methylase conferring resistance to erythromycin and clindamycin, was identified in 15/34 isolates (44.1%). In contrast, *ermA* and *linA* were not detected in any isolates. A single isolate (1/34, 2.9%) carried the macrolide efflux pump gene *msrA*. Aminoglycoside resistance genes were observed at low to moderate frequencies: *aacA-aphD* and *aadD* were each present in 4/34 isolates (11.8%), while *aphA3* and *sat* were detected in 1/34 isolates (2.9%) (Table 2). The trimethoprim resistance gene *dfrA* was detected in 6/34 isolates (17.6%) (Table 2).

The ribosomal protection gene *tetM* was detected in 18/34 isolates (52.9%) and the efflux gene *tetK* in 2/34 isolates (5.9%). Overall, 20/34 isolates (58.8%) carried at least one tetracycline resistance gene (Table 2). Phenicol resistance determinants were also frequent: *fexA*, encoding a chloramphenicol/florfenicol exporter, was present in 18/34 isolates (52.9%), while *cat* was not detected. The *fosB* gene, encoding a metallothiol transferase associated with fosfomycin resistance, was highly prevalent and detected in 30/34 isolates (88.2%), although *fosB*-plasmid variants were absent (Table 2).

The fusidic acid resistance gene *fusC* was identified in 14/34 isolates (41.2%), while *far1*, vancomycin resistance genes (*vanA*, *vanB*), and the mupirocin resistance gene *mupA* were absent from all isolates. Quaternary ammonium compound resistance genes (*qacA/qacC*) were detected in 2/34 isolates (5.9%) (Table 2).

### 3.3 Virulence and enterotoxin gene profiles

The distribution of virulence and enterotoxin genes is presented in Table 3. PVL genes (*lukF-PV* and *lukS-PV*), associated with severe skin and soft tissue infections, were detected in 2/34 isolates (5.9%). The *tst1* gene, encoding toxic shock syndrome toxin 1, was found in a single isolate (2.9%). No isolates (0/34) carried exfoliative toxin genes (*etA*, *etB*, *etD*), epidermal cell differentiation inhibitor genes (*edinA*, *edinB*, *edinC*), or the ACME locus (Table 3).

**Table 3:** Detection of virulence and enterotoxin genes among MRSA isolated from retail chicken meat in the United Arab Emirates.

		No. positive	% positive
Virulence genes			
*tst1*	Toxic shock syndrome toxin 1	1	3
*lukF-PV; lukS-PV*	Panton Valentine leukocidin F/S component	2	6
*sak*	Staphylokinase	32	94
*chp*	Chemotaxis-inhibiting protein	3	9
*scn*	Staphylococcal complement inhibitor	30	88
*etA*	Exfoliative toxin serotype A	0	0
*etB*	Exfoliative toxin serotype B	0	0
*etD*	Exfoliative toxin D	0	0
*edinA*	Epidermal cell differentiation inhibitor A	0	0
*edinB*	Epidermal cell differentiation inhibitor B	0	0
*edinC*	Epidermal cell differentiation inhibitor C	0	0
*ACME*	Arginine catabolic mobile element locus	0	0
*sasG*	*Staphylococcus aureus* surface protein G	34	100
*icaA*	Intercellular adhesion protein A	33	97
*icaC*	Intercellular adhesion protein C	34	100
*icaD*	Intercellular adhesion protein D	33	97
*bap*	Surface protein involved in biofilm formation	0	0
*clfA*	Clumping factor A	34	100
*clfB*	Clumping factor B	34	100
*can*	Collagen-binding adhesin	14	41
*fnbA*	Fibronectin-binding protein A	34	100
*fnbB*	Fibronectin-binding protein B	34	100
Enterotoxins genes			
*sea*	Enterotoxin A	12	35
*seb*	Enterotoxin B	1	3
*sec*	Enterotoxin C	1	3
*sed*	Enterotoxin D	14	41
*see*	Enterotoxin E	0	0
*seh*	Enterotoxin H	1	3
*sej*	Enterotoxin J	14	41
*sek*	Enterotoxin K	2	6
*sel*	Enterotoxin L	1	3
*seq*	Enterotoxin Q	2	6
*ser*	Enterotoxin R	14	41
*seg*	Enterotoxin G	19	56
*sei*	Enterotoxin I	18	53
*selm*	Enterotoxin M	19	56
*seln*	Enterotoxin N	16	47
*selo*	Enterotoxin O	12	35
*selu*	Enterotoxin U and/or Y	19	56

Immune evasion cluster genes were widely distributed. The staphylokinase gene *sak* was detected in 32/34 isolates (94.1%), while the staphylococcal complement inhibitor gene *scn* was present in 30/34 isolates (88.2%). The chemotaxis-inhibiting protein gene *chp* was found in 3/34 isolates (8.8%). These patterns are consistent with the predominance of human-adapted MRSA lineages (Table 3).

Adhesion and biofilm-associated genes were almost universally present. All isolates carried *sasG* (surface protein G), *clfA* and *clfB* (clumping factors A and B), and *fnbA* and *fnbB* (fibronectin-binding proteins A and B) (Table 3). Intercellular adhesion genes *icaA* and *icaD* were detected in 33/34 isolates (97.1%), and *icaC* in all 34 isolates (100%). The biofilm-associated surface protein gene *bap* was not detected. The collagen-binding adhesin gene *cna* was present in 14/34 isolates (41.2%) (Table 3).

A broad spectrum of staphylococcal enterotoxin genes was identified. Components of the enterotoxin gene cluster—including *seg*, *selm*, *selu* and *sei*—were observed in high proportions of isolates: *seg*, *selm* and *selu* were each detected in 19/34 isolates (55.9%), and *sei* in 18/34 isolates (52.9%). Other enterotoxin genes such as *sed*, *sej* and *ser* were present in 14/34 isolates (41.2%). The *seln* gene was detected in 16/34 isolates (47.1%). Among classical staphylococcal enterotoxins, *sea* was the most prevalent, occurring in 12/34 isolates (35.3%), while *seb*, *sec*, *seh*, *sel* and *seq* were detected in a minority of isolates and *see* was not detected (Table 3). The complete virulence and enterotoxin gene profiles are summarized in Table 3.

## 4 Discussion

The prevalence of MRSA in retail chicken meat from the original survey has been reported and discussed previously (Habib et al., 2025a); the present study focuses specifically on the microarray-based molecular characterization of those isolates and their comparison with UAE clinical human MRSA data. The results show that the clonal and virulence gene profiles are consistent with MRSA lineages commonly associated with human populations and may suggest potential human-related contamination along the food chain. In particular, CC5 and CC6, carrying community-associated SCC*mec* types IV and V/VT and a rich accessory genome that includes multiple AMR and virulence genes. To our knowledge, this is the first microarray-based molecular characterization from retail chicken meat in the UAE.

The predominance of CC5 and CC6 among chicken meat MRSA isolates characterized in the present study is closely mirroring the leading clonal complexes (CC5, CC6, CC361, CC22, CC1, CC8) identified among 310 human clinical MRSA isolates collected across the UAE in 2022 (Boucherabine et al., 2025). Earlier genotyping work also highlighted CC5 and CC22, along with USA300-like CC8, as important contributors to MRSA infections in the country (Senok et al., 2020; Thomsen et al., 2023). This clonal convergence with clinical MRSA suggests that chicken meat MRSA in the UAE more likely reflects human-associated strain pools circulating in the community, rather than livestock-adapted LA-MRSA lineages like CC398 or CC9 that are prevalent in intensive livestock systems elsewhere (Ribeiro et al., 2018; Fetsch et al., 2021; Monecke et al., 2018). This pattern is consistent with recent WGS-based analysis of MRSA from UAE retail red meat, in which CC5 and CC6 also predominated and only a single CC398 isolate was detected (Habib et al., 2025b). In that study, genomic relatedness between red meat isolates and clinical MRSA suggested that contamination likely arose from human sources along the slaughterhouse, processing or retail continuum (Habib et al., 2025b). The present microarray findings in chicken meat extend this picture to poultry, reinforcing the notion that MRSA in the UAE food chain is primarily human-associated.

SCC*mec* typing provides additional insight into the likely origin of these strains. In the chicken meat collection, SCC*mec* types IV and V/VT—hallmarks of community-associated MRSA—were the predominant elements, and no SCC*mec* II or III was detected. Boucherabine et al. (2025) similarly reported predominance of SCC*mec* IV and V/VT among clinical MRSA, particularly in CC5, CC6 and CC22 isolates, with only a minor contribution from hospital-associated SCC*mec* II lineages. Together, these data underscore the leading role of community-associated MRSA in both clinical and foodborne contexts in the UAE and suggest that shared human-adapted lineages are capable of contaminating meat products at some point along the farm-to-fork continuum.

The microarray revealed a broad accessory resistome among chicken meat MRSA isolates, with universal *mecA*, high prevalence of *bla* genes, *fosB*, *tetM*, *fexA* and frequent *ermC* and *fusC* carriage. These findings complement phenotypic resistance patterns reported in the original retail survey, where high levels of resistance to *β*-lactams, tetracyclines and fluoroquinolones were recorded among meat-derived MRSA isolates (Habib et al., 2025a). The genetic profile observed here is generally consistent with the antimicrobial resistance trends described for clinical MRSA in the UAE, which show broad multidrug resistance and rising complexity of resistance determinants over time (Thomsen et al., 2023; Boucherabine et al., 2025; Senghore et al., 2026).

Compared to UAE clinical MRSA (Boucherabine et al., 2025), chicken meat isolates showed higher carriage of *fusC* (41.2% vs. 13%, mainly CC5) and *ermC* (44.1% vs. ~ 25%). Conversely, *ermC* was identified in approximately one-quarter of clinical isolates but in 44.1% of chicken meat isolates (Boucherabine et al., 2025). Tetracycline resistance determinants are common in both settings, consistent with the widespread use of tetracyclines in food animals globally and their frequent association with mobile elements that circulate between human and livestock reservoirs (Ribeiro et al., 2018; Fetsch et al., 2021; Silva et al., 2021; Qiao et al., 2020).

Regionally, studies from poultry meat in Egypt, Iran and Algeria have reported high-level resistance to tetracyclines, macrolides, fluoroquinolones and β-lactams among MRSA isolates, with frequent detection of *mecA*, *tetM*, *tetK*, *erm* genes and various aminoglycoside resistance determinants (Sallam et al., 2015; Rahimi and Shafiei, 2019; Abolghait et al., 2020; Achek et al., 2021). The present UAE chicken meat isolate collection, dominated by human-associated lineages, shows a similar pattern of multidrug resistance but with a distinct emphasis on *fosB*, *fexA* and *fusC* in addition to *tetM* and *ermC*. This profile overlaps with clinical MRSA genotypes described in the Arabian Gulf region, including Saudi Arabia and neighbouring countries, where complex resistance patterns and SCC*mec*-encoded and plasmid-mediated determinants are prevalent (Saleem et al., 2025; Al-Saleh et al., 2022 cited in Thomsen et al., 2023).

Virulence profiling of the chicken meat MRSA isolates revealed widespread carriage of adhesion and biofilm-associated genes (*sasG*, *clfA*, *clfB*, *fnbA*, *fnbB*, *icaA*, *icaC*, *icaD*) and immune evasion cluster genes (*sak*, *scn*, *chp*). These gene combinations are characteristic of human-adapted MRSA lineages and are relevant for colonization of mucosal surfaces, immune evasion and persistence on food-contact surfaces (Miao et al., 2017; Chen et al., 2020; Abdullahi et al., 2021). The near-universal presence of major biofilm-associated genes (*ica* locus, *clfA/B*, *fnbA/B*) is consistent with strains capable of forming biofilms that could persist on food processing surfaces.

PVL genes (*lukF-PV/lukS-PV*) and *tst1* were detected in a small proportion of isolates (5.9 and 2.9%, respectively), in line with the lower frequency of these high-virulence markers among food-derived MRSA compared with certain clinical CA-MRSA lineages (Boucherabine et al., 2025; Achek et al., 2021). In the UAE clinical microarray study, *pvl* was present in 36% of isolates, and *tst1* in multiple CC22, CC30, and CC361 variants (Boucherabine et al., 2025). The lower PVL and *tst1* prevalence in chicken meat isolates may reflect differences in clinical versus food-associated strain composition; nevertheless, the detection of these genes in poultry meat, even at low frequency, is noteworthy from a One Health standpoint.

Of particular relevance to food safety is the extensive carriage of staphylococcal enterotoxin genes. Many chicken meat isolates in this study carried enterotoxin gene cluster components (*seg*, *sei*, *selm*, *seln*, *selu*) as well as classical enterotoxin genes such as *sea* and *sed*. Similar patterns have been reported in poultry meat MRSA from Egypt and Iran, where enterotoxin-positive strains were common and often associated with *sea*, *sed* and *seg* (Sallam et al., 2015; Rahimi and Shafiei, 2019; Abolghait et al., 2020). Globally, enterotoxin-positive MRSA in meat has been implicated in staphylococcal food poisoning outbreaks and is recognized as a major hazard in ready-to-eat foods (Benkerroum, 2018; Wendlandt et al., 2013; Nunes Nascimento et al., 2026).

The enterotoxin profiles observed in this study broadly resemble those detected in the recent UAE WGS-based analysis of MRSA from retail red meat, where a mixture of classical and new enterotoxin genes was identified across human-associated CC5 and CC6 backgrounds (Habib et al., 2025b). The present findings suggest that poultry meat may act as a vehicle for exposure to MRSA strains carrying enterotoxin genes. However, the foodborne risk implications must be interpreted with caution, since the presence of enterotoxin genes does not by itself confirm toxin expression, and staphylococcal food poisoning depends on bacterial growth and toxin production under favorable conditions in food (Nunes Nascimento et al., 2026). Although toxin expression and contamination levels at the point of consumption were not assessed here, these data support consideration of enterotoxin gene carriage in food-safety hazard identification for poultry products in the UAE.

When situated within the wider literature, the UAE poultry meat MRSA profile shows both convergence and divergence from patterns reported in other regions. In many European and North American settings, poultry-associated MRSA often includes a substantial component of LA-MRSA CC398 and CC9 lineages, which have been documented in broiler flocks, poultry meat and farm workers (Ribeiro et al., 2018; Fetsch et al., 2021; Silva et al., 2021; Pirolo et al., 2019). These lineages typically harbour tetracycline resistance genes and can display distinct SCC*mec* structures, such as the variable SCC*mec* elements described in CC398 (Monecke et al., 2018). By contrast, in the present UAE chicken meat collection, classic LA-MRSA lineages were not detected, and human-associated CC5 and CC6 predominated.

Within the MENA region, studies from Egypt, Iran and Algeria have identified both human-associated and livestock-associated MRSA lineages in poultry meat and poultry products, along with high rates of AMR and enterotoxin gene carriage (Sallam et al., 2015; Rahimi and Shafiei, 2019; Achek et al., 2021). A meta-analysis by Ribeiro et al. (2018) reported that MRSA prevalence in poultry and poultry meat was highest in South America and the Eastern Mediterranean, with substantial heterogeneity across study designs and sampling frames. The present study adds UAE-specific microarray data to this regional picture, demonstrating that in the Emirati context, poultry meat MRSA appears to be driven by human-associated CCs, similar to the dynamics observed for red meat (Habib et al., 2025b) and clinical MRSA (Boucherabine et al., 2025). For a full structured comparison of findings from regional poultry meat and other animal-derived food studies, readers are referred to the review by Belhout et al. (2022) on MRSA and other methicillin-resistant staphylococci/mammaliicocci in Arab countries. That review provides a useful framework for comparing regional trends in lineage distribution, SCC*mec* types, AMR determinants, and virulence gene carriage, and therefore helps place the present UAE findings within the wider Arab-country context.

The dominance of human-associated lineages in the UAE food chain likely reflects the interplay of local husbandry practices, antibiotic usage patterns, slaughterhouse and processing hygiene, and the demographic characteristics of the workforce, which together shape opportunities for human-to-food contamination (Thomsen et al., 2023; Senghore et al., 2026; Abdullahi et al., 2021). Comparable patterns have been reported in some other settings where food-derived MRSA primarily mirrors clinical CC distributions, suggesting that contamination rather than livestock adaptation is the main driver of food-chain MRSA (Achek et al., 2021; Dancer et al., 2019). However, further upstream sampling at farm and processing levels would be needed to systematically assess the contribution of live poultry reservoirs versus post-slaughter contamination in the UAE.

The present study demonstrates the utility of DNA microarray genotyping as a practical tool for molecular surveillance of MRSA along the food chain. Compared with WGS, DNA microarrays offer shorter turnaround times and simpler data handling, attributes that are advantageous for routine surveillance in diagnostic and food control laboratories. However, microarrays are inherently limited to detecting novel resistance genes, resolving fine-scale phylogenetic relationships, and assessing mobile genetic element structure. Additionally, as with any probe-based molecular assay, false-negative results for individual loci cannot be completely excluded; however, the applied validation procedures and internal quality-control criteria were used to support the reliability of the reported profiles. WGS remains the gold standard for detailed evolutionary analyses, outbreak investigation and discovery of emerging resistance mechanisms (Dancer et al., 2019; Nunes Nascimento et al., 2026). The present work should therefore be viewed as complementary to recent WGS-based studies of MRSA in UAE clinical and retail red meat populations (Boucherabine et al., 2025; Habib et al., 2025b).

In practical terms, a tiered approach in which microarray genotyping is used for broad baseline surveillance of MRSA along the human–animal–food interface, with targeted WGS applied to selected isolates of particular epidemiological interest (e.g., unusual CCs, novel SCC*mec* configurations, high-virulence profiles), may offer a cost-effective strategy for One Health MRSA monitoring (Senok et al., 2020; Fetsch et al., 2021; Achek et al., 2021). The present study provides an example of such an approach applied to poultry meat in the UAE.

Despite highlighting important knowledge gaps, the present study has some limitations to acknowledge. Sampling was limited to retail chicken meat in Dubai, and no data were collected at farm or processing levels, nor were longitudinal dynamics assessed. The clinical and food-derived isolate collections are not contemporaneous, limiting direct comparisons of temporal trends. Furthermore, the microarray analysis provides qualitative information on gene presence but not on gene expression, toxin production or quantitative contamination levels on meat at point of consumption. These limitations mean that the current results should be interpreted as descriptive baseline data rather than as a direct assessment of consumer risk.

Nevertheless, by documenting the clonal and molecular characteristics of poultry-associated MRSA and demonstrating substantial overlap with clinical MRSA profiles, the present work provides a rational basis for integrating retail meat surveillance into broader MRSA monitoring programmes in the UAE. In particular, routine testing of poultry products for MRSA, combined with targeted molecular characterization of representative isolates, would enable tracking of clonal shifts, emergence of new resistance or virulence patterns and potential convergence between food and clinical reservoirs. Such an approach aligns with international calls to embed One Health principles in AMR surveillance and food safety governance (Algammal et al., 2020; Nunes Nascimento et al., 2026; Xing et al., 2025).

## 5 Conclusion

This study provides the first microarray-based molecular characterization of MRSA isolates recovered from retail chicken meat in the UAE. The results show that contamination is dominated by human-associated CC5 and CC6 lineages carrying community-associated SCC*mec* types IV and V/VT and extensive antimicrobial resistance and virulence gene repertoires, including a wide range of staphylococcal enterotoxin genes. When interpreted alongside recent clinical microarray data and WGS-based analyses of MRSA from UAE retail red meat, these findings indicate that human-adapted MRSA lineages circulate across clinical and food-chain compartments in the country. These baseline molecular data justify integrating retail poultry meat surveillance into UAE One Health MRSA monitoring programs.

## Data Availability

The original contributions presented in the study are included in the article/supplementary material, further inquiries can be directed to the corresponding authors.
